# Immune Response and Protective Efficacy of a Heterologous DNA-Protein Immunization with* Leishmania* Superoxide Dismutase B1

**DOI:** 10.1155/2017/2145386

**Published:** 2017-11-22

**Authors:** Abebe Genetu Bayih, Nada S. Daifalla, Lashitew Gedamu

**Affiliations:** ^1^Department of Biological Sciences, University of Calgary, Room 374, 2500 University Drive NW, Calgary, AB, Canada T2N 1N4; ^2^Department of Medical Parasitology, School of Biomedical and Laboratory Sciences, College of Medicine and Health Sciences, University of Gondar, P.O. Box 196, Gondar, Ethiopia; ^3^The Forsyth Institute, Cambridge, MA 02142, USA

## Abstract

Growing evidence shows that antioxidant proteins of* Leishmania* could be used as vaccine candidates. In this study, we report the efficacy of* Leishmania donovani* iron superoxide dismutase B1 (LdFeSODB1) as a vaccine antigen in BALB/c mice in a DNA-protein prime-boost immunization regimen in the presence or absence of murine granulocyte macrophage colony stimulating factor (mGMCSF) DNA adjuvant. The expression study confirmed that LdFeSODB1 is expressed in mammalian cells and mGMCSF fusion mediates the secretion of the recombinant protein. Heterologous immunization with LdFeSODB1 induced a strong antibody- and cell-mediated immune response in mice. Immunization triggered a mixed Th1/Th2 response as evidenced by the ratio of IgG2a to IgG1. Antigen-stimulated spleen cells from the immunized mice produced high level IFN-*γ*. Multiparametric flow cytometry data showed that immunization with LdFeSODB1 induced significantly higher expression of TNF-*α* or IL-2 by antigen-stimulated T cells. Eight weeks after* L. major* infection, immunization with the antigen shifted the immune response to a more Th1 type than the controls as demonstrated by IgG2a/IgG1 ratio. Moreover, IFN-*γ* production by antigen-stimulated spleen cells from immunized mice remained high. The footpad swelling experiment showed that immunization with LdFeSODB1 resulted in partial protection of mice from a high dose* L. major* infection.

## 1. Introduction

Leishmaniasis is a vector-borne disease caused by protozoan parasites under the genus* Leishmania*. It is transmitted by the bite of sandfly vectors of the genera* Phlebotomus* and* Lutzomyia*. The disease is manifested in three major clinical forms, visceral, cutaneous, and mucocutaneous leishmaniasis. Visceral leishmaniasis, also known as Kala-azar, is a deadly disease with a mortality of about 100% in untreated clinically overt cases, whereas cutaneous leishmaniasis, though the most common form, causes skin lesions that usually heal spontaneously [1]. According to the recent World Health Organization (WHO) report, leishmaniasis is distributed in 98 countries and three territories. The annual incidence of cutaneous and visceral leishmaniasis is approximately 1 million and 300,000, respectively. Annually, visceral leishmaniasis causes deaths in the range of 20,000 to 50,000.* Leishmania* and HIV share similar host cells of an infected person. Hence,* Leishmania*-HIV coinfection exerts synergistic deleterious effect on the host. HIV infection increases the susceptibility to visceral leishmaniasis, reduces efficacy of drug treatment, and increases the rate of relapse. Unfortunately, the distribution of HIV-*Leishmania* coinfection is expanding. As of 2013, 35 leishmaniasis endemic countries have reported HIV-*Leishmania* coinfection [2].

Although drugs are available to treat leishmaniasis, they suffer from variable efficacy in different endemic areas, high toxicity, and/or unaffordability to the people that are affected by the disease. In addition, high level resistance to the first-line drugs is reported in some of the endemic countries with high prevalence of visceral leishmaniasis [3, 4].

The development of effective and affordable vaccines is believed to be one of the best ways to fight neglected tropical diseases such as leishmaniasis that are affecting millions of disadvantaged and marginalized people in developing countries. Although the fact that some people who are cured of leishmaniasis develop life-long immunity to reinfection gives high hope to the feasibility of developing vaccines to leishmaniasis, no effective vaccine has been developed for general human use to date. In the past, several vaccines were tried in the form of live or attenuated parasites. Although live parasite vaccines showed high level efficacy, they come with serious side-effects where some of the immunized individuals develop a full-blown disease. On the other hand, subunit vaccines in the form of recombinant parasite proteins or DNA are generally safe. However, these vaccines are usually too weak to induce a strong protective immunity which necessitates the use of effective adjuvants [5–7]. As a result, only very few of the numerous subunit vaccine candidates have reached to clinical trial phase in humans [8–10]. Antioxidant proteins such as superoxide dismutases have been tested for vaccine candidates for a variety of infectious diseases such as leishmaniasis [11, 12], schistosomiasis [13], and brucellosis [14]. Superoxide dismutase (SOD) of* Leishmania* is involved in the parasite's defense against the toxic oxygen radicals produced by the host cells to fight the infection. By converting the highly toxic radical, superoxide anion, into hydrogen peroxide,* Leishmania* SOD triggers the parasite's antioxidant activity eventually allowing the parasite to evade the host's first-line of defense and proliferate inside macrophages. Superoxide dismutases of* Leishmania* are different from those of human SODs.* Leishmania* possesses iron superoxide dismutase (FeSOD), whereas humans have copper/zinc SOD (Cu/Zn SOD).* Leishmania* possesses two SOD genes, LFeSODA and LFeSODB. LFeSODA is a single copy gene, whereas LFeSODB is a two-copy gene, LFeSODB1 and LFeSODB2. LFeSODB1 and LeFeSODB2 are predominantly expressed in the amastigote and promastigote stages, respectively [15, 16]. The difference between* Leishmania* and human SODs can be exploited to develop a safe vaccine to combat leishmaniasis. In addition, previous studies by our group showed that* Leishmania* SODB1 is highly conserved among the different VL and CL causing* Leishmania* species [15, 17].

We have developed a subunit vaccine candidate using* Leishmania donovani* iron superoxide dismutase B1 (LdFeSODB1). Previously, we showed that immunization with recombinant LdFeSODB1 protein induces immune response and partially protects susceptible BALB/c mice from* L. major* challenge infection in cutaneous leishmaniasis infection model [12, 18]. A more recent study by Campos and colleagues [19] demonstrated that DNA/DNA immunization with SOD induces immune response in BALB/c mice and partially protects the mice from* L. amazonensis* cognate infection.

The objective of this study is to evaluate the immunogenicity and protective efficacy of* L. donovani* iron superoxide dismutase B1 (LdFeSODB1) against cutaneous leishmaniasis in* L. major* infection model in BALB/c mice. We followed a DNA-recombinant protein prime-boost immunization regimen. We also used fusion murine granulocyte macrophage colony stimulating factor (mGMCSF) and bacterial CpG ODN as adjuvant.

## 2. Materials and Methods

### 2.1. Animals and* Leishmania* Strains

Female BALB/c mice were purchased (Charles River Laboratories, QC, Canada) and were maintained under pathogen-free animal facility of the Department of Biological Sciences, University of Calgary, Canada.* Leishmania major* strain V1 (MHOM/IL/80/Friedlin) was kindly provided by Dr. Steven G. Reed, Infectious Disease Research Institute (IDRI) (WA, USA).

### 2.2. Cloning of* Leishmania donovani* Iron Superoxide Dismutase

*Leishmania donovani* iron superoxide dismutase B1 (LdFeSODB1) gene was cloned into two different plasmid vectors. For DNA vaccine, the coding region of the gene was cloned into a modified pcDNA plasmid with or without mGMSCF fusion DNA. A spacer fragment was placed between mGMCSF and LdFeSODB1 genes. The spacer region possesses six histidine residues for recombinant protein purification, an enteropeptidase cleavage site, and flanking proline residues at both ends. After PCR amplification, the LdFeSODB1 gene and also the plasmid were digested with* NotI-HF*® restriction enzyme (New England BioLabs, Canada). After ligation of the PCR product and the plasmids, transformation of* E. coli* DH5*α* was performed. Confirmation of the cloning was done by sequencing of plasmid DNA extracted from transformed* E. coli*.

Endotoxin-free vaccine candidate plasmid DNA was isolated from the transformed* E. coli* DH5*α* using EndoFree® plasmid purification kit (QIAGEN, Canada) following the manufacturer's instruction. Endotoxin-free plasmid DNA samples were diluted to appropriate concentration using endotoxin-free PBS (Teknova, USA) and were used for protein expression study in Chinese Hamster Ovary cells (CHO) and for injection into mice. For expression in bacterial system, LdFeSODB1 was cloned into pET17b plasmid (Novagen) following previously published procedure [18].

### 2.3. Transfection of Chinese Hamster Ovary Cells, Expression of Fusion Protein, and Western Blotting

Chinese Hamster Ovary cells (CHO) (Life Technologies, USA) were cultured in CD CHO medium (1x) (Gibco®) supplemented with HT supplement (Gibco) and L-glutamine (Gibco). CHO cells were transfected with the vaccine antigens pcDNA-LdFeSODB1 and pcDNA-mGMCSF-LdFeSODB1 as well as controls (pcDNA and pcDNA-mGMSCF). Transfection was also done using* pEGFPN3* (Clonetech, USA) as expression control. Cationic lipid-mediated transfection of CHO cells with DNA vaccine candidates was done using Lipofectamine® 2000 transfection reagent (Invitrogen) following the previously published procedure [11]. Transfection of CHO cells and expression of the DNA vaccine candidates were confirmed by Western blotting.

Western blotting was performed to check the expression of rLdFeSODB1 and fusion rmGMCSF-LdFeSODB1 as well as the secretion of these proteins as previously described [11]. Briefly, cell culture supernatant and whole cell lysate were used for Western blotting to assess the secretion of the recombinant proteins. Culture supernatant (SUP) and cell lysate (LYS) samples taken from CHO cells that were transfected with pcDNA, pcDNA-mGMCSF, pcDNA-LdFeSODB1, and pcDNA-mGMCSF-LdFeSODB1 as well as rmGMCSF control (AbCam, Canada) were loaded into 12% SDS-polyacrylamide gel and probed with rabbit anti-mGMCSF polyclonal antibody (AbCam, Canada) and mouse anti-LdFeSODB1 antiserum. Western blotting was performed following the instruction on ECL Western blotting detection system manual (Amersham GE Healthcare, UK).

### 2.4. Expression and Purification of Recombinant LdFeSODB1

LdFeSODB1 gene was expressed and the His-tagged recombinant LdFeSODB1 protein was purified from* E. coli* Tuner™ (DE3)* pLysS* cells using Ni-NTA agarose (QIAGEN) column chromatography. Briefly, the competent bacterial cells were transformed with the plasmid DNA containing LdFeSODB1 gene. Then, the bacteria were cultured in Luria-Bertani medium and the expression was induced with 2 mM IPTG (Isopropyl beta-D-thiogalactoside). The bacterial pellet was thawed and subjected to lysis with mild sonication in 20 mM Tris-Cl, pH 8.0 containing 1 mM PMSF and EDTA-free Complete Mini protease inhibitor cocktail tablet (Roche, Germany). The suspension was treated with 1% CHAPS (Sigma) in Tris-Cl and incubated for 4 hr in the cold room. Then, it was spun down and supernatant discarded. The protein was purified from the inclusion bodies. The pellet was resuspended in binding buffer containing 8 M urea (Sigma) in Tris-Cl followed by mixing it with 5 ml Ni-NTA agarose and incubation at room temperature for 1 hr on a rotating Compact Rocker (Mandel, Canada). The mixture was then transferred into Purification Column (Invitrogen), washed once with buffer containing 8 M urea, and then treated with 12 mM sodium deoxycholate (Sigma, USA) in 20 mM Tris-Cl pH 6.3 and followed by a series of washes with Tris-Cl pH 8.0 without urea. Finally, the rLdFeSODB1 was eluted with Tris-Cl pH 8.0 buffer containing 0.4 M imidazole (Sigma, USA). The rLdFeSODB1 protein was then dialyzed using 1x PBS, pH 9.0. Finally, endotoxin was removed using Detoxy-Gel AffinityPack prepacked columns (Pierce, USA). The rLdFeSODB1 protein was stored in −80°C freezer until use.

### 2.5. Immunization and Infection

Four- to -six-week-old female BALB/c mice were acclimatized for two weeks before immunization started. Five mice were randomly assigned to each of the vaccine groups and controls. DNA-protein immunization was performed with two doses of DNA immunization (pcDNA-LdFeSODB1 or pcDNA-mGMCSF-LdFeSODB1) followed by a single booster immunization with rLdFeSODB1 each given in three-week interval. DNA immunization was performed by intramuscular injection of a mixture of 100 *μ*g plasmid DNA and 25 *μ*g CpG ODN 1826* (5*′*-tccatgacgttcctgacgtt-3*′) (InvivoGen, USA) dissolved in a total 50 *μ*l endotoxin-free PBS (Teknova, USA). The recombinant protein booster immunization was given to the vaccine groups by subcutaneous injection (SC) of 12.5 *μ*g rLdFeSODB1 protein in combination with 25 *μ*g CpG ODN in the right hind footpad. All the three injections to the mice that received pcDNA or pcDNA-mGMCSF controls were given in the form of plasmid DNA only combined with CpG ODN. In addition, two control groups of mice were included that were given three injections of CpG ODN only or PBS.

*Leishmania major* strain V1 (MHOM/IL/80/Friedlin) was used for the protection study in BALB/c mice. Preparation of the parasite and mice infection was done using previously published protocol [11]. Briefly, stationary phase promastigotes were washed and 3 × 10^6^ live parasites in 40 *μ*l endotoxin-free PBS were injected subcutaneously into the hind left footpad of each mouse three weeks after the last immunization. The thickness of the footpads was then measured every week until euthanasia using an electronic digital caliper (VWR, USA). Mice that had a net footpad swelling of more than 3 mm thick and/or those that developed necrotic lesions were euthanized.

### 2.6. Blood Collection, Spleen Cell Isolation, and Stimulation

Blood samples were collected every week by retro-orbital sinus bleeding and at the time of euthanasia by cardiac puncture. Serum was isolated from whole blood samples and stored at −20°C freezer until used.

Mouse spleen was collected aseptically and cells were isolated from each mouse separately as described previously [12]. Cells were washed with cRPMI and seeded at 2 × 10^5^ cells per well in 100 *μ*l medium in triplicate in a 96-well tissue culture plate (Sarstedt, USA). Then, the cells from individual mouse were stimulated with ConA (5 *μ*g/ml), recombinant LdFeSODB1 protein (10 *μ*g/ml), or* L. major* SLA (50 *μ*g/ml) separately. Cells in the unstimulated group received medium alone. The cells were incubated for 72 hr at 37°C and 5% carbon dioxide (CO_2_). At 72 hr, culture supernatant was transferred into a new plate, sealed, and stored in −80°C freezer until cytokine ELISA was done.

### 2.7. Measurement of Antibody Response

The magnitude of antigen-specific antibody response was assessed by measuring rLdFeSODB1-specific mouse total IgG, IgG1, and IgG2a antibody in sera from each mouse that was immunized with the vaccine antigens or controls using indirect enzyme-linked immunosorbent assay (ELISA). Ninety six-well flat-bottom Nunc MaxiSorp ELISA plates (eBiosciences, USA) were coated with 10 *μ*g/ml rLdFeSODB1 in 50 *μ*l/well bicarbonate buffer (pH 9.6) and incubated overnight at 4°C. Blocking, washing, and detection were performed following previously published procedure [11].

### 2.8. Measurement of Cytokine Response

The cell-mediated immune response of vaccinated mice and controls was assessed by measuring the level of interferon-gamma (IFN-*γ*) and IL-10 in culture supernatant of antigen/mitogen stimulated and unstimulated spleen cells using BD OptEIA™ Set Mouse IFN-*γ* and BD OptEIA Set Mouse IL-10 (BD Biosciences, USA) kits, respectively. The absorbance was read on a microplate reader (Molecular Devices, USA) and the concentration of the cytokines in the sample was calculated against the concentration of the standard using SoftMax Pro 5 software (Molecular Devices, USA). The cytokines were measured from stimulated spleen cells that were isolated from individual mouse.

### 2.9. Intracellular Cytokine Staining and Flow Cytometry

To further dissect the cell-mediated immune response, a seven-color flow cytometry was performed on stimulated spleen cells using a three-laser BD FACSAria II machine. All reagents for intracellular staining and flow cytometry were purchased from BD Biosciences (CA, USA). The detailed procedure of the flow cytometry was described previously [11]. Briefly, 1 × 10^6^ cells in 100 *μ*l cRPMI per well were stimulated with phorbol myristate acetate (PMA) (5 ng/ml)/ionomycin (500 ng/ml) (Sigma), 10 *μ*g/ml rLdFeSOB1 protein antigen, 50 *μ*g/ml* L. major* SLA or medium alone (unstimulated). The cells were surface-stained with V450 rat anti-mouse CD3, V500 rat anti-mouse CD4, and APC-Cy7 rat anti-mouse CD8*α*. The cells were then washed 2 times with staining buffer. After fixation and permeabilization, blocking, and washing, the cells were stained with PE-Cy7 rat anti-mouse IFN-*γ*, FITC rat anti-mouse TNF-*α*, PE rat anti-mouse IL-2, and APC rat anti-mouse IL-10. Isotype control staining was done on antigen-stimulated cells (in separate wells) with equal concentration of isotype-matched control of irrelevant specificity. For unstained control, antigen-stimulated cells were treated with staining buffer devoid of any antibody. After washing twice, the cells were resuspended in PBS and analyzed using BD FACSAria II machine. Compensation was done using equivalent mixture of BD™ CompBeads Anti-Rat Ig, *κ*, and BD CompBeads Negative Control (FBS) following the manufacturer's instruction. The result was analyzed using FlowJo software (Tree Star, Inc, USA). The lymphocytes were gated based on the size, granularity, and surface and intracellular staining profiles. The flow cytometry was done on spleen cells that were isolated from each mouse separately and the result was expressed as the mean ± standard error of the mean (SEM).

### 2.10. Statistical Analysis

All the tests were done on individual mouse samples and the mean ± standard error of the mean (SEM) of five mice per group was compared with the respective control. The statistical differences between different groups of mice were analyzed using Kruskal-Wallis test, whereas the difference between means of any two groups was compared using Mann–Whitney *U* test. A *p* value of less than 0.05 was considered statistically significant. Statistical analysis was done using IBM SPSS Statistics 20 software.

### 2.11. Ethics Statement

The experimental protocol for the mice study (BI 2006-33) was reviewed and approved by the Life and Environmental Sciences Animal Care Committee (LESACC), The University of Calgary. The experiments were done in accordance with the principles by The Canadian Council on Animal Care. Mice were monitored twice daily throughout the study period and a veterinarian was consulted as required. To minimize suffering, all injections were performed under anesthesia using isoflurane inhalation. The mice that developed a net footpad swelling of more than 3 mm thick and/or necrotic lesion were euthanized. Mice were sacrificed by carbon dioxide inhalation. There was no unexpected death of mice.

## 3. Results

### 3.1. Expression of the Vaccine Antigen in Mammalian Cells

**Figure 1:**
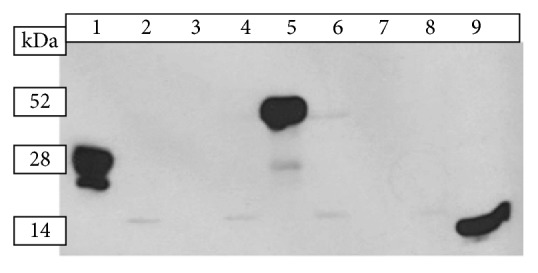
*Western blotting of samples from CHO cells transfected with LdFeSODB1 gene cloned in pcDNA and pcDNA-mGMCSF*. Cell culture supernatant (SUP) and cell lysate (LYS) proteins of transfected CHO cells were run on 12% denaturing polyacrylamide gel and Western blotting was done using rabbit-anti-mGMCSF and ECL-anti-rabbit IgG-HRP (donkey) primary and secondary antibodies, respectively and Lanes: (1) pcDNA-mGMCSF-SUP, (2) pcDNA-mGMCSF-LYS, (3) pcDNA-LdFeSODB1-SUP, (4) pcDNA-LdFeSODB1-LYS, (5) pcDNA-mGMCSF-LdFeSODB1-SUP, (6) pcDNA-mGMCSF-LdFeSODB1-LYS, (7) pcDNA-SUP, (8) pcDNA-LYS, and (9) recombinant mGMCSF protein.

Confirmation of expression of the vaccine antigen in mammalian cells is a prerequisite for any DNA vaccine to be used in animal models and consequently in humans. We also investigated the secretion of the vaccine antigen by transfected mammalian cells. CHO cells were transfected using the plasmids carrying the vaccine antigens and the expression of LdFeSODB1 and the fusion mGMCSF-LdFeSODB1 were analyzed. Western blotting using rabbit anti-mGMCSF polyclonal primary antibody demonstrated that the fusion mGMCSF-LdFeSODB1 is expressed in mammalian cells and secreted out of the cell (Figure 1). Culture supernatant samples from cells that were transfected with pcDNA-mGMCSF and pcDNA-mGMCSF-LdFeSODB1 showed bands of about 25 to 28 KDa and 52 KDa, respectively. The control rmGMCSF protein showed a band of about 14 KDa. However, there was no signal in samples taken from cells that were transfected with pcDNA-LdFeSODB1 (Figure 1).

### 3.2. LdFeSODB1 Induces Antigen-Specific Antibody Response in Mice

**Figure 2:**
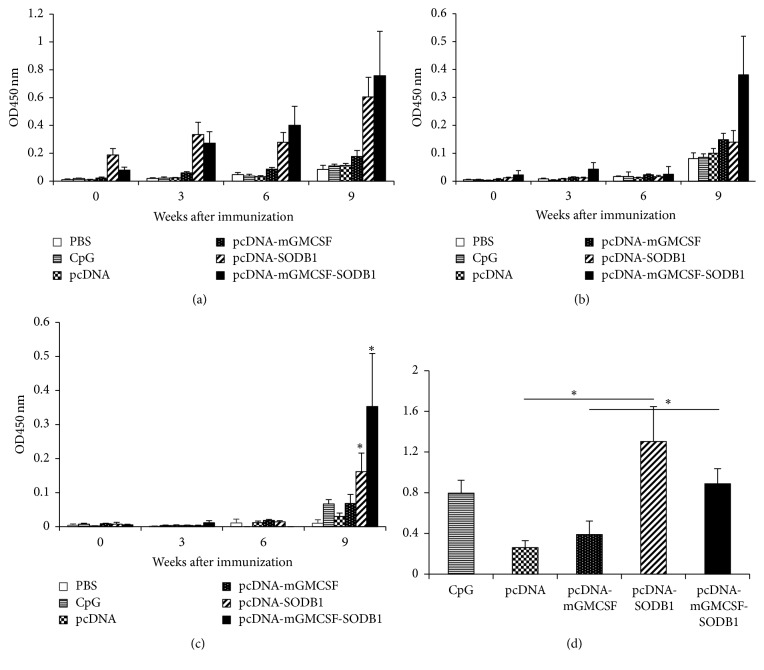
*Antigen-specific antibody response in BALB/c mice before challenge*. Mice were immunized twice with LdFeSODB1 DNA antigens and controls followed by a boost with the recombinant LdFeSODB1 protein. All immunizations were given in three-week intervals. Blood samples were collected before immunization, at the time of each immunization and upon euthanasia. Total IgG (a), IgG1 (b), and IgG2a (c) against rLdFeSODB1 were measured using ELISA and the result is depicted as mean OD_450 nm_ of five mice per group and standard error of the mean (SEM). The mean IgG2a/IgG1 ratio is depicted in (d). Statistical comparison between groups was performed using Mann–Whitney *U* test. The assay was done in duplicate wells for each mouse serum. This is one of two experiments with similar result. The asterisk shows statistically significant difference between the vaccine antigen and the respective control groups (*p* < 0.05).

Immunization with LdFeSODB1 in the presence and absence of mGMCSF induces antigen-specific antibody response in mice (Figure 2). As seen in week 6 after immunization response, injection with two doses of pcDNA-LdFeSODB1 and pcDNA-mGMCSF-LdFeSODB1 DNA antigens elicits significantly higher total IgG response than the respective controls, pcDNA and pcDNA-mGMCSF (*p* < 0.05). Similarly, the antigen immunized mice produce significantly higher total IgG than the controls after rLdFeSODB1 protein boost (week 9) (*p* < 0.05) (Figure 2(a)).

To analyze the type of antibody response, we measured antigen-specific IgG1 and IgG2a response. As shown in Figures 2(b) and 2(c), the antigen in the presence or absence of mGMCSF induced a mixed IgG1 and IgG2a response. There was no significant difference between the vaccine groups and the controls with regard to the production of IgG1. However, the vaccine groups produced significantly higher IgG2a response than the control groups at week 9 after immunization (*p* < 0.05) (Figure 2(c)). At week 9, the mean IgG2a/IgG1 ratios in mice that received pcDNA-LdFeSODB1, pcDNA-mGMCSF-LdFeSODB1, pcDNA, and pcDNA-mGMCSF were 1.3 ± 0.34, 0.89 ± 0.15, 0.26 ± 0.07, and 0.39 ± 0.13, respectively. The difference in IgG2a/IgG1 ratio between the mice that were immunized with the vaccine antigen and that of the respective controls was statistically significant (*p* < 0.05) (Figure 2(d)).

### 3.3. Cell-Mediated Immune Response before Challenge Infection

**Figure 3:**
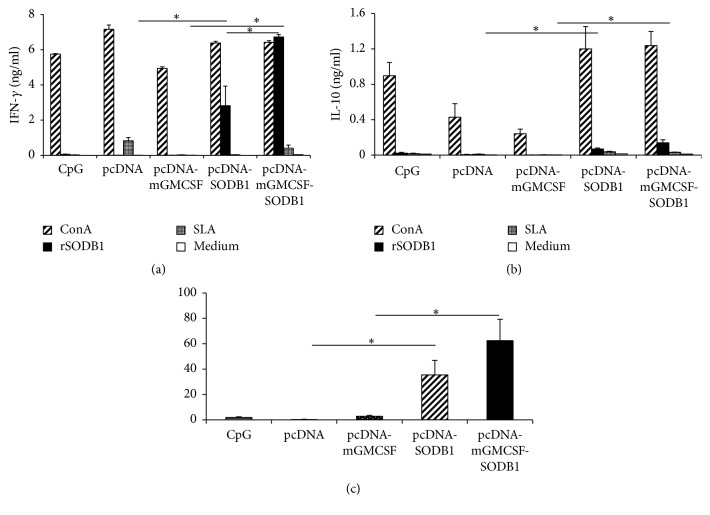
*Prechallenge cytokine response of mice immunized with LdFeSODB1 antigen*. The level of cytokine production was assessed in stimulated spleen cells. (a) IFN-*γ*, (b) IL-10, and (c) mean IFN-*γ*/IL-10 ratio. Mice were immunized twice with pcDNA-LdFeSODB1 or pcDNA-mGMCSF-LdFeSODB1 and boosted with rLdFeSODB1 protein. The control mice received three doses of pcDNA, pcDNA-mGMCSF, or CpG ODN alone. The level of IFN-*γ* and IL-10 was measured from stimulated spleen cells using cytokine ELISA kit (BD Biosciences). The concentration of the cytokines (ng/ml) was calculated by correlating the optical density to concentration of the protein standard included in the kit. The mean concentration and standard error of the mean (SEM) of five mice per group are shown. This is one of two experiments with similar result. The IFN-*γ*/IL-10 ratio was calculated from cells that were stimulated with rLdFeSODB1. Statistical comparison between groups was performed using Mann–Whitney *U* test. Asterisks indicate statistically significant difference in the cytokine production between mice immunized with antigen and the respective controls (*p* < 0.05).

The degree of cell-mediated immune response elicited in mice immunized with the vaccine antigens was assessed by measuring the level of IFN-*γ* and IL-10 in antigen-stimulated spleen cell. Spleen cells isolated from mice that were immunized with pcDNA-LdFeSODB1 and pcDNA-mGMCSF-LdFeSODB1 and stimulated with rLdFeSODB1 produced significantly higher IFN-*γ* and IL-10 than those that received the controls pcDNA and pcDNA-mGMCSF, respectively (*p* < 0.05). In other words, antigen-stimulated cells from the control mice produced barely detectable IFN-*γ* and IL-10 (Figures 3(a) and 3(b)). On the other hand, mice that were immunized with pcDNA-mGMCSF-LdFeSODB1 antigen produced significantly higher IFN-*γ* than those that received the antigen without fusion mGMCSF (*p* < 0.05). However, the difference in the level of IL-10 was not statistically significant. Stimulation of the spleen cells with SLA did not produce detectable level of cytokines in all mice groups (Figures 3(a) and 3(b)).

The IFN-*γ*/IL-10 ratios were 35.4 ± 11.5, 62.4 ± 16.8, 0.2 ± 0.2, and 2.8 ± 0.73, for pcDNA-LdFeSODB1, pcDNA-mGMCSF-LdFeSODB1, pcDNA, and pcDNA-mGMCSF immunized mice, respectively. The difference between the antigen immunized mice and the respective controls was statistically significant (*p* < 0.05) (Figure 3(c)).

### 3.4. Phenotype of Prechallenge Antigen-Specific Cytokine Producing CD4^+^ and CD8^+^ T Cells

**Figure 4:**
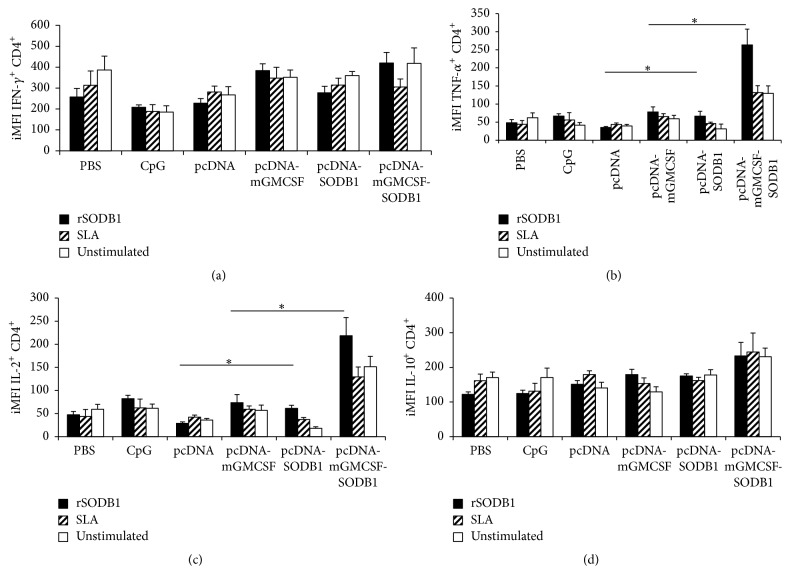
*Antigen-specific cytokine expressing *CD4^+^* T cells three weeks after the last immunization*. Integrated median fluorescent intensity (iMFI) of IFN-*γ* (a), TNF-*α* (b), IL-2 (c), and IL-10 (d). Integrated MFI was calculated as a product of the percentage of the cytokine producing CD4^+^ T cells and the median fluorescent intensity of the cytokine. Mice immunized twice with pcDNA-LdLdFeSODB1 or pcDNA-mGMCSF-LdFeSODB1 were boosted with rLdFeSODB1 protein. The control mice received three doses of pcDNA, pcDNA-mGMCSF, or CpG ODN alone. The percentage of cytokine producing CD4^+^ T cells as well as MFI was measured in stimulated spleen cells from individual mice. The mean iMFI and standard error of the mean (SEM) of five mice per group are shown. Asterisks indicate statistically significant difference between cells from antigen immunized mice and controls (*p* < 0.05). Stimulation of spleen cells with PMA/ionomycin produced consistently high response in all groups (data not shown).

Multiparametric flow cytometry was performed to analyze the phenotype of cytokine producing T cells in spleen cells isolated from mice that were immunized with the vaccine antigen and the controls. To understand the full magnitude of cytokine response using flow cytometry, we calculated the Integrated Median Fluorescent Intensity (iMFI) by multiplying the percentage of cytokine expressing T cells and the MFI [20]. Spleen cells that were stimulated with PMA resulted in high level of cytokines in all groups. As shown in Figure 4, spleen cells from mice immunized with pcDNA-LdFeSODB1 and pcDNA-mGMCSF-LdFeSODB1 showed significantly higher iMFI for CD4^+^TNF-*α* and CD4^+^IL-2 than the controls pcDNA and pcDNA-mGMCSF, respectively (*p* < 0.05). However, the vaccine groups did not show significantly higher iMFI for IFN-*γ* and IL-10 than the controls.

**Figure 5:**
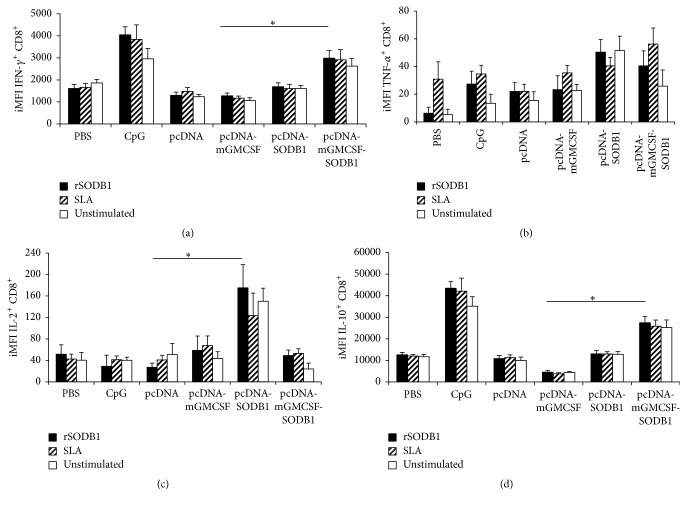
*Antigen-specific cytokine expressing *CD8^+^* T cells three weeks after the last immunization*. Integrated median fluorescent intensity (iMFI) of IFN-*γ* (a), TNF-*α* (b), IL-2 (c), and IL-10 (d). Integrated MFI was calculated as a product of the percentage of the cytokine producing CD8^+^ T cells and the median fluorescent intensity of the cytokine. Mice immunized twice with pcDNA-LdLdFeSODB1 or pcDNA-mGMCSF-LdFeSODB1 were boosted with rLdFeSODB1 protein. The control mice received three doses of pcDNA, pcDNA-mGMCSF, or CpG ODN alone. The percentage of cytokine producing CD8^+^ T cells as well as MFI was measured in stimulated spleen cells from individual mice. The mean iMFI and standard error of the mean (SEM) of five mice per group are shown. Asterisks indicate statistically significant difference between cells from antigen immunized mice and controls (*p* < 0.05). Stimulation of spleen cells with PMA/ionomycin produced consistently high response in all groups (data not shown).

Generally, antigen-stimulated spleen cells that were isolated from mice that received the vaccine antigens contained more CD8^+^ cells that express the cytokines tested than the cells from control mice (Figure 5). The mice that were immunized with pcDNA-LdFeSODB1 induced significantly higher CD8^+^ IL-2^+^ than control mice (*p* < 0.05). On the other hand, immunization with the antigen in the presence of mGMCSF resulted in significantly higher iMFI for CD8^+^ IFN-*γ*^+^ and CD8^+^ IL-10^+^ than the respective control, pcDNA-mGMCSF (*p* < 0.05) (Figures 5(a) and 5(d)). Unexpectedly, the cells from mice that received CpG ODN adjuvant only also showed high level iMFI for CD8^+^ IFN-*γ*^+^ and CD8^+^ IL-10^+^.

### 3.5. Postchallenge Antibody Response

**Figure 6:**
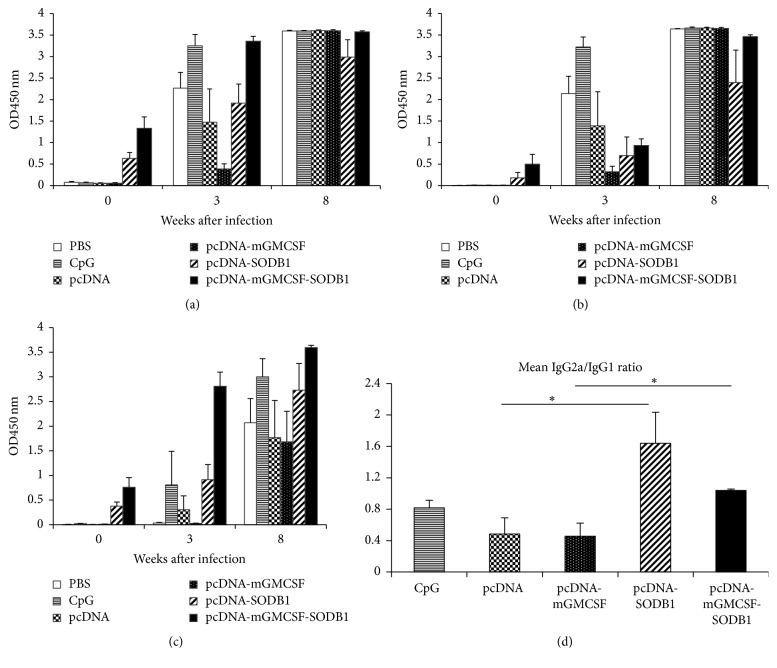
*Antigen-specific antibody response in BALB/c mice after L. major infection*. LdFeSODB1-specific total IgG (a), IgG1 (b), IgG2a (c), and IgG2a/IgG1 ratio; (d) antibody response after infection with* L. major*. The antibody response was measured using ELISA and the result is depicted as mean OD_450 nm_ of five mice per group and standard error of the mean (SEM). The assay was done in duplicate wells for each mouse serum. Point 0 indicates the time when the mice were infected with* L. Major.* The statistical difference was compared using compared using Mann–Whitney *U* test. Asterisks indicate statistically significant difference in IgG2a/IgG1 ratio between mice immunized with the antigen and the control groups (*p* < 0.05).

To investigate the durability of the immune response after challenge infection with* L. major*, we measured the level of antibody response three and eight weeks after infection. As expected, we found antigen-specific total IgG response both in the mice immunized with the vaccine antigens and also in the control groups (Figure 6(a)). Likewise, we found that the mice that were immunized with the vaccine antigens and those that received the control preparations showed antigen-specific IgG1 and IgG2a response (Figures 6(b) and 6(c)). However, the IgG2a/IgG1 ratio varied greatly between the antigen immunized mice and the controls. At week 8 after infection, the IgG2a/IgG1 ratios in serum samples from mice that received pcDNA-LdFeSODB1, pcDNA-mGMCSF-LdFeSODB1, pcDNA, and pcDNA-mGMCSF were 1.64 ± 0.39, 1.04 ± 0.02, 0.48 ± 0.2, and 0.46 ± 0.17, respectively. The difference between the vaccine groups and their respective controls was statistically significant (*p* < 0.05) (Figure 6(d)).

### 3.6. Postchallenge Cytokine Response

**Figure 7:**
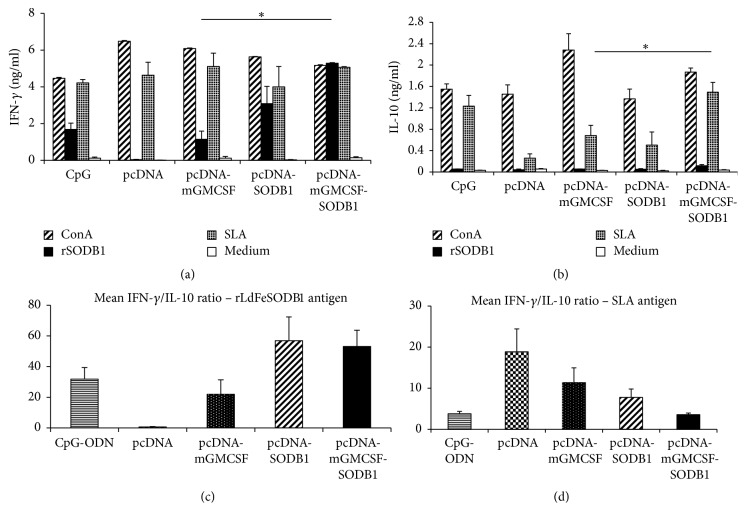
*Postchallenge cytokine response of mice immunized with LdFeSODB1 antigen*. IFN-*γ* (a) and IL-10 (b) production in stimulated spleen cells was measured using cytokine ELISA. Mice immunized twice with pcDNA-LdFeSODB1 or pcDNA-mGMCSF-LdFeSODB were boosted with rLdFeSODB1. The control mice received three doses of pcDNA, pcDNA-mGMCSF, or CpG ODN alone. Mice were infected with stationary phase* L major* three weeks after the last immunization. Cytokine ELISA was done on antigen-stimulated and control spleen cells isolated from mice at eight weeks after infection. The data represent the mean cytokine concentration of five mice per group. The mean IFN-*γ*/IL-10 ratios in cells stimulated with rLdFeSODB1 and SLA are depicted in (c) and (d), respectively. Statistical comparison between groups was performed using Mann–Whitney *U* test. Asterisks indicate statistically significant difference in the cytokine production between mice immunized with antigen and the respective control (*p* < 0.05).

Like the antibody response, we also investigated the durability of cell-mediated response after infection and whether the vaccine antigens are targets of immune response after infection. This was done by measuring the level of IFN-*γ* and IL-10 from culture supernatant of spleen cells that were stimulated with rLdFeSODB1 and SLA. Upon stimulation with rLdFeSODB1, cells from mice that were immunized with the antigen in the presence or absence of mGMCSF produced high level IFN-*γ*. However, only the mice that were immunized with pcDNA-mGMCSF-LdFeSODB1 showed statistically significant difference with the control group, pcDNA-mGMCSF (*p* < 0.05) (Figure 7(a)). Stimulation of the cells with SLA produced high level IFN-*γ* in the vaccinated groups and controls alike but there was no significant difference in the level of IFN-*γ* between any two groups (Figure 7(a)). On the other hand, both the vaccinated and control groups produced very low level IL-10 on stimulation with rLdFeSODB1. However, stimulation of the cells with SLA produced appreciable amount of IL-10 in the experimental and control groups. As compared with the respective control group, mice immunized with pcDNA-mGMCSF-LdFeSODB1 produced significantly higher IL-10 (*p* < 0.05) (Figure 7(b)). The IFN-*γ*/IL-10 ratios in rLdFeSODB1 stimulated cells were 56.9 ± 15.5, 53.2 ± 10.6, 0.68 ± 0.24, and 22.0 ± 9.4 for mice that received pcDNA-LdFeSODB1, pcDNA-mGMCSF-LdFeSODB1, pcDNA, and pcDNA-mGMCSF, respectively. However, the difference between the antigen immunized groups and the controls was not statistically significant (Figure 7(c)). On the other hand, stimulation of the spleen cells from pcDNA-mGMCSF-LdFeSODB1 mice with SLA showed the least IFN-*γ*/IL-10 ratio. However, the difference was not statistically significant (Figure 7(d)).

### 3.7. Footpad Swelling

**Figure 8:**
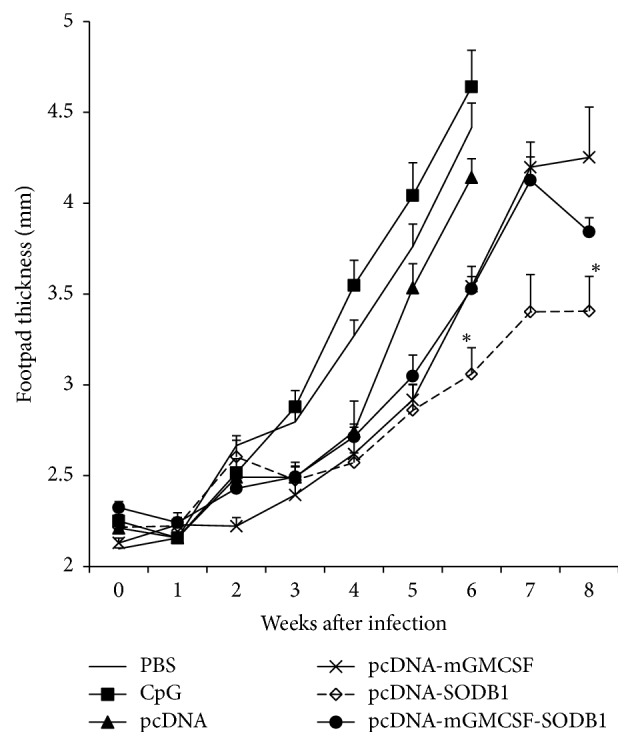
*Footpad swelling of mice immunized with antigens and controls in DNA/protein immunization strategy and infected with L. major*. BALB/c mice (five per group) were immunized twice with DNA antigens and controls in the presence of CpG ODN adjuvant followed by a boost with recombinant LdLdFeSODB1 protein. All immunizations were given in three-week intervals. At week 9, all mice were infected on the footpad with subcutaneous injection of 3 × 10^6^ stationary phase* L. major* promastigotes in the hind footpad. The footpad swelling was assessed by measuring the thickness of infected footpad weekly for eight weeks using electronic digital caliper. The data represents mean footpad size in millimetre of five mice and standard error of the mean. Asterisks indicate statistically significant difference in footpad swelling between antigen immunized mice and controls (*p* < 0.05).

The protective efficacy of LdFeSODB1 was evaluated by comparing the footpad swelling of the mice that were immunized with the vaccine antigens and those of controls after* L. major* infection. Three million stationary phase live promastigotes were injected to the footpad of each mouse and the swelling was measured by electronic metric caliper every week. As shown in Figure 8, mice from most of the control groups were sacrificed early at week 6 after infection due to development of too big footpad lesion and/or necrosis. Generally, the mice that were immunized with the vaccine antigen with or without mGMCSF fusion adjuvant showed smaller footpad swelling than most of the control groups. At week 6 after infection, mice immunized with pcDNA-LdFeSODB1 developed significantly smaller footpad than the control mice that received pcDNA control (*p* < 0.05). However, the footpad swelling of the mice immunized with the antigen with mGMCSF fusion did not show any difference from those that received pcDNA-mGMCSF control. At week 8 after infection, mice infected with the vaccine antigen generally developed smaller footpad swelling than the respective controls. However, the difference was significant only in pcDNA-LdFeSODB1 immunized mice. These mice developed significantly smaller footpad at week 8 than the mice that received pcDNA control at week 6 (*p* < 0.05).

## 4. Discussion

Development of an effective and safe* Leishmania* vaccine has been considered as an attainable endeavour to effectively prevent and control the spread of leishmaniasis and prevent the devastating impact of visceral leishmaniasis in endemic countries where the very poor and disadvantaged segment of the population are disproportionately affected. The observation that some infected people develop a life-long immunity to reinfection was the source of the optimism. Unfortunately, however, no effective human vaccine has been developed so far despite the trial of numerous candidate vaccines in animal models. The reason is multifold: (1) most of the subunit vaccines are too weak to induce a strong and durable immune response, (2) there is lack of proper and safe adjuvant to be used together with the subunit vaccines in humans, (3) there is lack of defined and universally accepted correlates of protection, and (4) there is a difference between the human and mouse immune system [6, 7]. In light of these shortcomings, we have developed a DNA vaccine antigen that is fused with GMCSF to develop a single antigen-adjuvant combination for induction of a specific and durable immune response.

Our group has been studying the possible use of antioxidant proteins of* Leishmania* as possible subunit vaccine candidates in the form of recombinant protein and/or DNA. We have also studied the use of different adjuvants together with the vaccine antigens [11, 12, 18, 21].

In this study, we investigated the efficacy of LdFeSODB1 as cutaneous leishmaniasis vaccine candidate in a DNA-protein prime-boost immunization regimen in mice in the presence of mGMCSF fusion adjuvant. We showed previously that immunization with LdFeSODB1 induces a cross-reactive response to* L. major*. Western blotting experiment showed that antiserum raised using* L. donovani* FeSODB1 reacted with whole cell lysate of both* L. donovani* and* L. major* and recombinant LdFeSODB1 [12]. Moreover, Yeganeh and colleagues [22] found that* L. major* SODB1 is recognized by immune sera of CL and VL patients. Hence, LdFeSODB1 antigen could be used as a vaccine candidate for both visceral and cutaneous leishmaniasis.

The purpose of fusing mGMCSF to LdFeSODB1 gene was twofold. By its immunomodulatory effect, mGMCSF serves as an adjuvant. Secondly, mGMCSF protein has a leader sequence that mediates the secretion of fused vaccine candidate proteins out of the myocytes upon intramuscular injection making the proteins available for dendritic cells for antigen presentation [23]. GMCSF has been used as an adjuvant in a variety of candidate vaccines [24, 25].

As a prerequisite, we checked the expression of the DNA vaccine antigen in mammalian cells* in vitro*. We showed that the fusion mGMCSF-LdFeSODB1 is expressed in CHO cells. The secreted fusion mGMCSF-LdFeSODB1 protein that we detected in our experiment ran at a higher molecular size than expected. Together with the peptide of the spacer sequence, the expected mGMCSF-LdFeSODB1 was 36 KDa. However, a band of about 52 KDa was seen in the supernatant of cells that were transfected with pcDNA-mGMCSF-LdFeSODB1. Similar size increment was seen in cells that were transfected with pcDNA-mGMCSF. This result is in agreement with other studies which showed similar size discrepancy that involved the expression of GMCSF in mammalian cells [23, 26, 27]. The difference from the expected size is attributed to glycosylation of GMCSF in mammalian cells.

The detection of the majority of rmGMCSF-LdFeSODB1 in the cell supernatant confirmed the secretion role of mGMCSF. Unfortunately, we could not show the expression of the antigen in the absence of mGMCSF in CHO cells. Several Western blotting experiments on the lysate and supernatant samples taken from transfected CHO cells using mouse anti-LdFeSODB1 antibody failed to show a visible signal. This could be because the low titer of the mouse anti-LdFeSODB1 antiserum was not strong enough to detect the rLdFeSODB1 protein from transfected CHO cells. Increasing the concentration of the antiserum produced too much background that we could not get a specific signal.

Previous studies have shown that DNA vaccines induce all forms of specific immune response, antibody-mediated as well as CD4^+^- and CD8^+^-mediated response. The CD8^+^ response is especially important to induce protective immune response against intracellular infections such as* Leishmania* [28, 29]. Early work of Gurunathan and colleagues [29] demonstrated that DNA vaccine formulation is superior to recombinant protein form. Another study also showed that* Leishmania* DNA vaccine elicits both CD4^+^- and CD8^+^-mediated immune responses [28]. However, DNA vaccines generally produce weaker immune response in humans than in small animals [30, 31]. Therefore, we used a heterologous DNA/protein immunization method so as to utilize the benefit of both DNA and recombinant protein vaccine formulations. A more recent study has also shown that a heterologous DNA/protein immunization induces more effective and protective response than DNA/DNA or protein/protein regimens [32]. As expected, administration of a single dose of rLdFeSODB1 protein boost three weeks after the second injection with the DNA vaccine dramatically increased the specific antibody response. As compared with protein-protein immunization with rLdFeSODB1 [12, 18], the DNA-protein immunization in this study induced higher cell-mediated immunity as seen in high IFN-*γ* and IL-10 production by antigen-stimulated spleen cells.

*Leishmania major* infection in mice is characterized by induction of T-helper-1 (Th-1) and T-helper-2 (Th-2) immune response. Early studies have shown that the balance between Th-1 and Th-2 determines the outcome of the infection. Induction of Th-1 immune response with production of IFN-*γ*, TNF-*α*, and IL-2 is associated with resistance to* L. major* infection. By producing IFN-*γ* and TNF-*α*, Th-1 cells activate macrophages to kill intracellular pathogens such as* Leishmania* and also help CD8^+^ T cells to execute their cytotoxic function. On the other hand, induction of a Th-2 immune response with the production of cytokines such as IL-4, IL-10, and IL-13 is associated with suppression of anti-*Leishmania* activity of macrophages and results in the development of severe disease [33–35]. Therefore, the type and magnitude of prechallenge cytokine response elicited by vaccine antigens is used to measure the efficacy of the vaccine in protecting against* L. major* infection. In addition, the magnitude of IgG2a and IgG1 antibody response is used as indirect indication of the quality of immune response after immunization. The production of IFN-*γ* and IL-4 induce isotype switching to IgG2a and IgG1 phenotypes, respectively [36]. Thus, the ratio of IgG2a to IgG1 is considered as an indirect indication of the quality of the immune response. IgG2a/IgG1 ratio equal to one generally indicates that the antigen induced a mixed immune response with equivalent level of Th1 and Th2 phenotypes. Increase in the ratio is an indication of the level of shift of the immune response into a more Th1 type. Conversely, a reduction of the ratio indicates the shift to a more Th2 type of response [37, 38]. Based on the IgG2a/IgG1 ratio, LdFeSODB1 with or without mGMCSF fusion adjuvant induced a mixed Th1/Th2 response with a shift to more Th1 phenotype than the controls. Although the antigen without mGMCSF showed slightly higher ratio than the antigen with fusion mGMCSF, the difference is not statistically significant. The IgG2a/IgG1 ratio of the vaccine groups remained higher than the controls eight weeks after infection with* L. major*. Serum samples from infected control mice resulted in IgG2a/IgG1 ratio of about 0.5 showing that immunization with the vaccine antigens shifted the immune response to a more Th-1-biased phenotype than the controls. In other words, the increase in IgG2a/IgG1 ratio in postinfection serum samples that were obtained from antigen immunized mice compared to the samples obtained from mice that received the controls shows the power of the antigen in shifting the default immune response of a predominantly Th2 phenotype in the control mice (with a ratio of less than 1) to a more Th1 phenotype. Studies have shown that the level of IgG2a/IgG1 ratio is generally a reflection of the level of resistance to or protection from* L. major* infection and is correlated with parasite load and/or footpad swelling in infected mice [38–40].

Production of high level IFN-*γ* before infection is generally considered as an indicator of protective potential of a vaccine candidate against* L. major* infection [29]. Compared to the control groups, antigen-stimulated spleen cells isolated from mice that were immunized with LdFeSODB1 with or without mGMCSF produced significantly high level IFN-*γ* before infection indicating the induction of Th-1 response. Unfortunately, we could not measure IL-4 production from stimulated cells due to technical problem. However, we measured the level of IL-10. Studies have shown that the protective potential of a vaccine candidate depends on not only the level of IFN-*γ* but also the level of IL-10 [41]. IL-10 plays crucial role in susceptibility of mice to* L major* infection. It favors disease progression by inhibiting the development of Th-1 cells and by blocking activation of macrophage by IFN-*γ* [7]. Therefore, we calculated the prechallenge ratio of IFN-*γ* to IL-10 to assess the protective potential of a vaccine antigen. Immunization with LdFeSODB1 in the presence or absence of mGMCSF resulted in significantly higher IFN-*γ* to IL-10 ratio than the controls (*p* < 0.05). Although the ratio remained high eight weeks after* L. major* infection, there was no statistically significant difference between the antigen groups and the controls. The IFN-*γ* to IL-10 ratio obtained in antigen-stimulated spleen cells in DNA/protein immunization in this study is more than 15 times greater than the ratio obtained in our previous study involving protein/protein immunization [18].

In order to further dissect the phenotype of antigen-specific T cells, we performed multiparametric flow cytometry. This allowed us to thoroughly assess the production of Th-1 cytokines, IFN-*γ*, TNF-*α*, and IL-2 by CD4^+^ and CD8^+^ T cells. It appears that the source of IFN-*γ* in antigen-stimulated spleen cells of mice immunized with pcDNA-LdFeSODB1 is different from that of cells from pcDNA-mGMSCF-LdFeSODB1 immunized mice. In spleen cells of mice immunized with pcDNA-LdFeSODB1, the IFN-*γ* might have been produced by cells different from T cells. Moreover, it appears that high level IL-2 was produced by CD8^+^ cells in addition to the amount produced by CD4^+^ cells in pcDNA-LdFeSODB1 immunized mice. In mice that were immunized with pcDNA-mGMCSF-LdFeSODB1 antigen, IL-2 appears to be produced mainly by CD4^+^ T cells. On the other hand, antigen-stimulated CD8^+^ cells from pcDNA-mGMCSF-LdFeSODB1 produced higher level IL-10.

The protective efficacy of the antigen was measured by measuring footpad swelling after a high dose subcutaneous infection with* L. major*. The footpad swelling result in antigen immunized mice appears to be loosely correlated with the IgG2a/IgG1 ratio before and after parasite challenge and IFN-*γ*/IL-10 ratio before infection. The level of IL-10 produced by SLA stimulated spleen cells after parasite challenge is also correlated with the footpad swelling data. That is, the mice that were immunized with the antigen in the presence of mGMCSF produced higher IL-10 response than the mice that were immunized with the antigen devoid of mGMCSF. However, the data do not show a clear correlation between the level of protection and the expression of Th-1 cytokines by antigen-stimulated CD4^+^ and CD8^+^ T cells. Interestingly, although the use of mGMCSF adjuvant with LdFeSODB1 increased the immunogenicity with higher production of antigen-specific antibodies and also cytokines such as IFN-*γ*, it did not bring proportionally higher protection against* L. major* infection. This is in stark contrast with our previous study with LdPxn1 where the mGMCSF fusion increased both the immunogenicity and protective efficacy of the antigen [11]. The possibility of the different role of GMCSF when used together with different antigens needs further study. The major limitation of this study was the absence of parasite load data. Our attempt to measure parasite load from the footpad of immunized mice and controls was not successful due to technical problem. It would be interesting to see if the preinfection immunogenicity data of mice immunized with the antigen in the presence of mGMCSF is better correlated with parasite load reduction.

## 5. Conclusion

We have shown that DNA-protein immunization with LdFeSODB1 elicited a strong antibody and cell-mediated immune response in BALB/c mice. The antigen also induced partial protection against subcutaneous infection with* L. major*. Although the presence of mGMCSF fusion adjuvant significantly increased the immunogenicity of LdFeSODB1 antigen, it did not proportionally increase the protective efficacy of the antigen. It would be interesting to investigate the protective potential of the antigen in a low-dose infection model in mice.
